# A short synthetic peptide fragment of human C2ORF40 has therapeutic potential in breast cancer

**DOI:** 10.18632/oncotarget.16713

**Published:** 2017-03-30

**Authors:** Chaoyang Li, Pengju Zhang, Anli Jiang, Jian-Hua Mao, Guangwei Wei

**Affiliations:** ^1^ Department of Biochemistry and Molecular Biology, Shandong University School of Medicine, Jinan, Shandong, 250012, P.R. China; ^2^ Department of Human Anatomy and Key Laboratory of Experimental Teratology, Ministry of Education, Shandong University School of Medicine, Jinan, Shandong, 250012, P.R. China; ^3^ Biological Systems and Engineering Division, Lawrence Berkeley National Laboratory, Berkeley, CA 94720, USA

**Keywords:** C2ORF40 mimic peptide, breast cancer, proliferation, xenograft, mitosis

## Abstract

C2ORF40 encodes a secreted protein which is cleaved to generate soluble peptides by proteolytic processing and this process is believed to be necessary for C2ORF40 to exert cell type specific biological activity. Here, we reported a short mimic peptide of human C2ORF40 acts potential therapeutic efficacy in human cancer cells *in vitro* and *in vivo*. We synthesized a short peptide of human C2ORF40, named C2ORF40 mimic peptide fragment and assessed its biological function on cancer cell growth, migration and tumorigenesis. Cell growth assay showed that C2ORF40 mimic peptide fragment significantly suppressed cell proliferation of breast and lung cancer cells. Moreover, C2ORF40 mimic peptide fragment significantly inhibited the migration and invasion of breast cancer cells. Furthermore, we showed that this peptide suppressed tumorigenesis in breast tumor xenograft model. Cell cycle assay indicated that the C2ORF40 mimic peptide fragment suppressed the growth of tumor cells through inducing mitotic phase arrest. In conclusion, our results firstly suggested that this short synthetic peptide of human C2ORF40 may be a candidate tumor therapeutic agent.

## INTRODUCTION

C2ORF40 (chromosome 2 open reading frame 40), also named esophageal cancer related gene 4 (ECRG4) or proaugurin, is firstly found by Su *et al*. in esophageal cancer [1]. In recent years, researchers have found that C2ORF40 is a tumor suppressor gene, which exists multiple functions on the cell proliferation, migration and cell senescence [2–4]. Our previous study indicated that DNA hypermethylation of the C2ORF40 promoter could downregulate its transcript level in human breast cancer cells [5]. The downregulation of C2ORF40 was found in various malignant tumors including esophageal tumor [6, 7], prostate tumor [8, 9], colorectal carcinoma [2] and breast cancer [5], and the lower expression of this gene was significantly correlated with worse survival rate [10, 11]. Restoration of C2ORF40 expression could significantly suppress proliferation, migration and invasion of breast and other cancer cells [5, 12–15]. These data indicated that restoration of C2ORF40 could serve as therapeutic potential in human malignant tumors.

Unlike other prototypic tumor suppressors, C2ORF40 resembles a neuropeptide-like precursor, which is characteristic of the human secretome [16]. C2ORF40 gene encodes a 148 amino acid protein with multiple cleavage sites which could be identified by the furin or thrombin-like enzymes and processed to generate multiple small peptides [17–20]. Indeed, these small soluble peptides were detected in the cell medium and conditioned cell culture supernant could suppress cell proliferation [2, 20, 21]. Further investigations indicate that C2ORF40 itself could not suppress cell proliferation and processing of C2ORF40 protein is required to suppress proliferation of tumor cell lines [22]. It is reported a thrombin processed C2ORF40-derived 16 amino acid peptide itself has functional roles, it provide the possibility that delivery of this small peptide could have therapeutic potential [20, 23–25]. However, whether this thrombin processed C2ORF40-derived small peptide could involve in the regulation of breast cancer progression has not been investigated.

In the present study, we synthesized this 16 amino acid peptide, named as C2ORF40 mimic peptide fragment (C2ORF40MPF) and assessed its biological function on breast and other cancer cells based on the clinical significance in comparison of C2ORF40 full length to explore its therapeutic potential. We first confirmed that loss of C2ORF40 protein expression was found correlated with the clinicopathologic characteristics of human breast cancer using our clinical samples and restoration of C2ORF40 arrested cancer cell cycle progression at M phase by cell cycle synchronization techniques. Functional analysis of this synthesized C2ORF40MPF showed that it could suppress the proliferation and migration of breast and lung cancer cells *in vitro* and the tumorigenesis in breast tumor xenograft model. Our results indicated that this C2ORF40MPF may be a candidate tumor therapeutic agent by mimicking the restoration of C2ORF40 expression.

## RESULTS

### C2ORF40 protein expression deficiency correlates with breast cancer clinicopathologic characteristics

**Figure 1:**
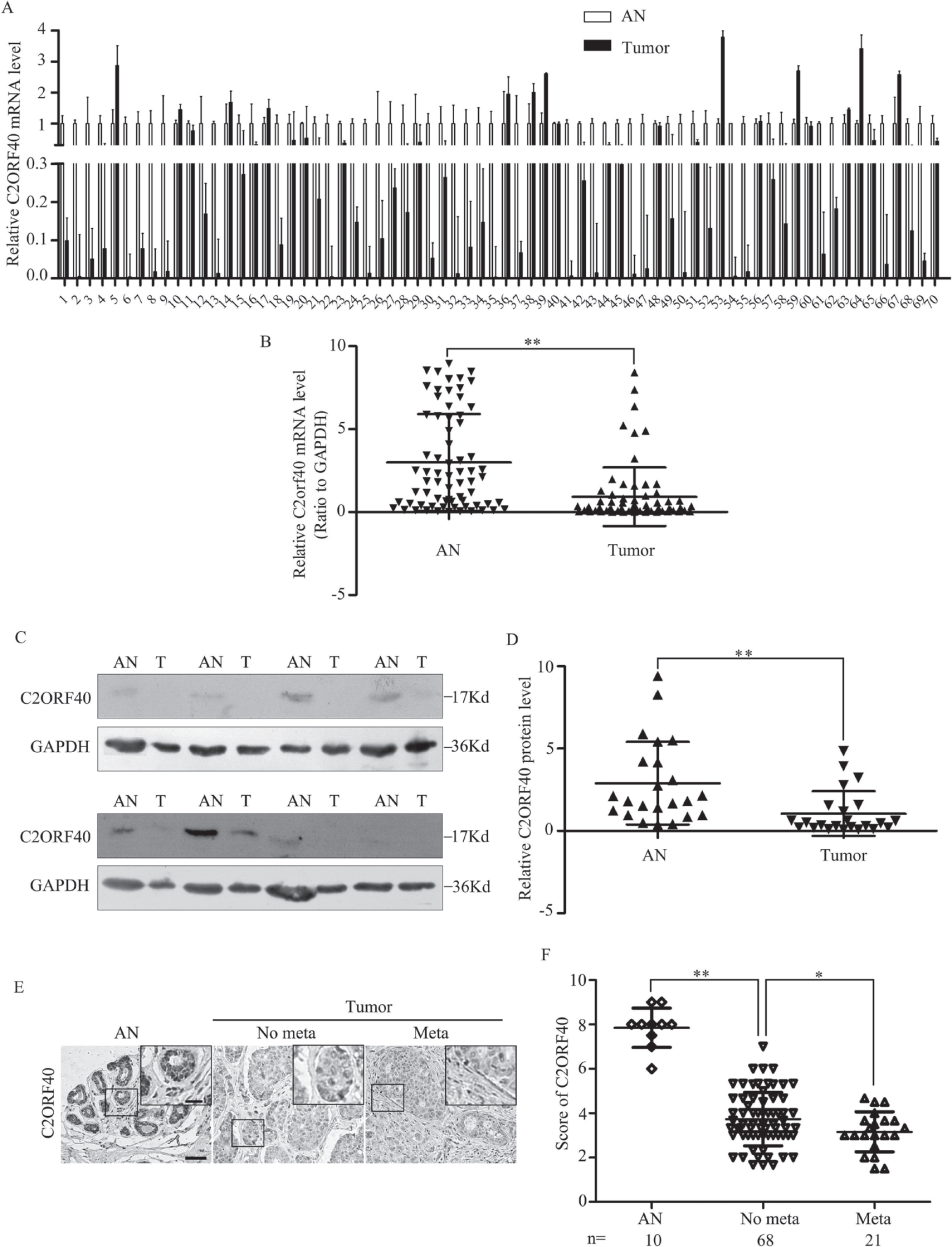
C2ORF40 expression deficiency correlates with breast cancer clinicopathologic characteristics (**A** and **B**) qRT-PCR analysis of C2ORF40 mRNA expression levels in human breast cancer and adjacent nontumor tissues. *n* = 70. *GAPDH* mRNA was used as the control. AN, adjacent nontumor tissues; T, tumor tissues. (**C**) Western blotting analysis of C2ORF40 protein level in human breast cancer and adjacent nontumor tissues. Representative results were shown. GAPDH protein was used as the control. AN, adjacent nontumor tissues and T, tumor tissues. (**D**) Relative C2ORF40 protein expression levels in breast cancer and adjacent noncancer tissues (C2ORF40/GAPDH, *n* = 23). (**E**) Representative images of C2ORF40 IHC staining in primary cancer tissues with or without metastasis and corresponding tumor adjacent nontumor tissues. (**F**) Corresponding semiquantification of C2ORF40 expression was shown. AN, adjacent nontumor tissue; No meta, primary cancers without metastasis; Meta, primary cancers with metastasis. Scale bars: 50 μm (E) and 20 μm (insets in E). *P < 0.05 and **P < 0.01 based on the Student *t* test. Data are represented as mean ± SD.

We first evaluated C2ORF40 protein expression in the breast cancer tissues from our tissue banks to verify the clinical significance of C2ORF40 protein. Transcriptional analysis showed that the C2ORF40 mRNA level was significantly lower in 58 out of 70 breast cancer tissues (Figure 1A and 1B, Supplementary Figure 1A), which is consistent with our GEO data analysis [5]. We then determined the protein level of C2ORF40 in 23 paired primary breast cancer tissues and corresponding non-cancerous tissues by Western blotting assay. We found a highly significant deficiency of C2ORF40 expression in all the breast cancer samples (*P* < 0.01) (Figure 1C and 1D), which was consistent with the qRT-PCR results.

To define the clinical significance of C2ORF40 in breast cancer, immunohistochemical (IHC) staining was performed in a breast tissue array. IHC staining confirmed the expression of C2ORF40 were normal in nontumorous breast tissue, lower in primary breast cancer and lowest in breast cancer with metastasis (Figure 1E and 1F). Correlation analysis of C2ORF40 protein expression with clinicopathologic features revealed significant association between deficiency of C2ORF40 expression and TNM stage, metastasis and differentiation (Figure 1E and 1F; Table 1), indicating the involvement of C2ORF40 deficiency in breast cancer progression. These data demonstrated the closely correlation between C2ORF40 protein expression deficiency and the clinicopathologic characteristics of human breast cancer.

**Table 1:** Correlation between C2ORF40 expression and clinicopathological characteristics of breast tumors

Clinicopathologicalparameters	NO.of samples	IHC score	*P*
Age (years)	< 60 70 ≧ 60 19	3.65 ± 1.20 3.35 ± 1.00	*P* > 0.05^a^
Tumor with metastasis No Yes	68 21	3.73 ± 1.21 3.15 ± 0.90	*P* < 0.05^a^
Differentiation Normal I II III	10 5 62 16	7.85 ± 0.88 5.60 ± 0.89 3.49 ± 1.06 4.90 ± 1.46	*P* < 0.01^b,1^
TNM stage Normal I II III	10 5 67 17	8.35 ± 1.00 4.82 ± 1.30 3.55 ± 1.13 3.48 ± 1.14	*P* < 0.01^b,2^

^a^Student *t* test; ^b^one-way ANOVA.

^1^Differences between grade I and III; II and III are not significant.

^2^Differences between stage I and III; II and III are not significant.

### Synthesized C2ORF40 mimic peptide fragment inhibits the growth of breast cancer cells

**Figure 2:**
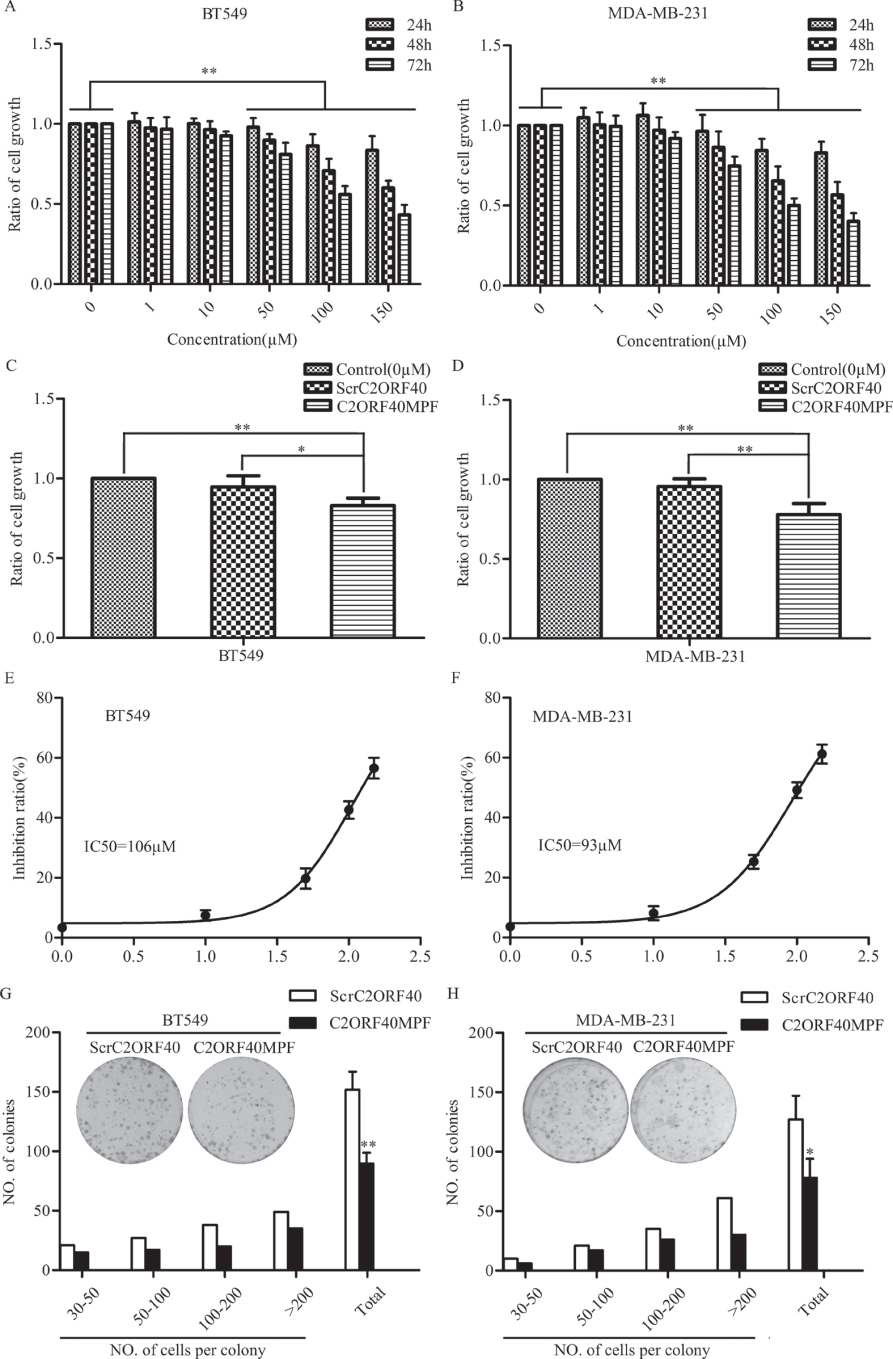
The effect of C2ORF40MPF on the growth of human breast cancer cells (**A**) BT549 and (**B**) MDA-MB-231 cells were treated with the indicated concentrations of C2ORF40MPF for 24, 48, and 72 hours. Cell proliferation *in vitro* was examined by MTT assay. (**C** and **D**) Cells were treated with 75 μM ScrC2ORF40 or C2ORF40MPF. Cell proliferation *in vitro* was examined by MTT assay. IC50 of (**E**) BT549 and (**F**) MDA-MB-231 were calculated using GraphPad Prism5 from representative experiments and the peptide concentration were plotted as the Log [M]. (**G** and **H**) Colony formation assay demonstrated a significant decrease in the number of colonies by treatment with C2ORF40 mimic peptide in BT549 and MDA-MB-231 cells. ScrC2ORF40 stands for Scrambled C2ORF40 mimic peptide, while C2ORF40MPF for C2ORF40 mimic peptide fragment. All results are from at least three independent experiments. *P < 0.05 and **P < 0.01 based on the Student *t* test. Data are represented as mean ± SD.

We previously reported that C2ORF40 has tumor suppressor function by inhibiting cancer cell proliferation [5]. *C2ORF40* gene encodes a 148 amino acid protein which could be proteolytically processed and secreted as smaller peptides [2, 17, 19]. To better elucidate the role of C2ORF40 in breast cancer, we synthesized a 16 amino acid peptide derived from C-terminal domain of human C2ORF40 (Supplementary Figure 4), named as C2ORF40 Mimic Peptide Fragment (C2ORF40MPF). To examine if this synthetic C2ORF40MPF could function equivalently as C2ORF40 in breast cancer cells, we assessed its inhibitory ability on cell growth using MTT assay, and used non-treatment and the synthesized scrambled C2ORF40 mimic peptide (ScrC2ORF40) as controls. Firstly, we examined the time and dose dependence of this C2ORF40MPF on cell growth. The results indicated that C2ORF40MPF significantly inhibited breast cancer cell viability in a time- and dose-dependent manner (Figure 2A and 2B), while ScrC2ORF40 mimic peptide did not inhibit the viability of breast cancer cells compared with non-treatment control (Figure 2C and 2D). In addition, in Figure 2E and 2F, we calculated the IC50 of this peptide in BT549 (IC50=106 μM) and MDAMB231 (IC50=93 μM) cells. In colony formation assay, treatment with C2ORF40MPF significantly decreased the numbers and sizes of clones in BT549 (Figure 2G) and MDA-MB-231 (Figure 2H) cells. Furthermore, lung cancer cell viability was also inhibited by C2ORF40MPF in a time- and dose-dependent manner (Supplementary Figure 2A and 2B). Therefore, we concluded that C2ORF40MPF could inhibit breast and lung cancer cell viability.

### C2ORF40MPF inhibit the migration and invasion of human breast cancer cells

**Figure 3:**
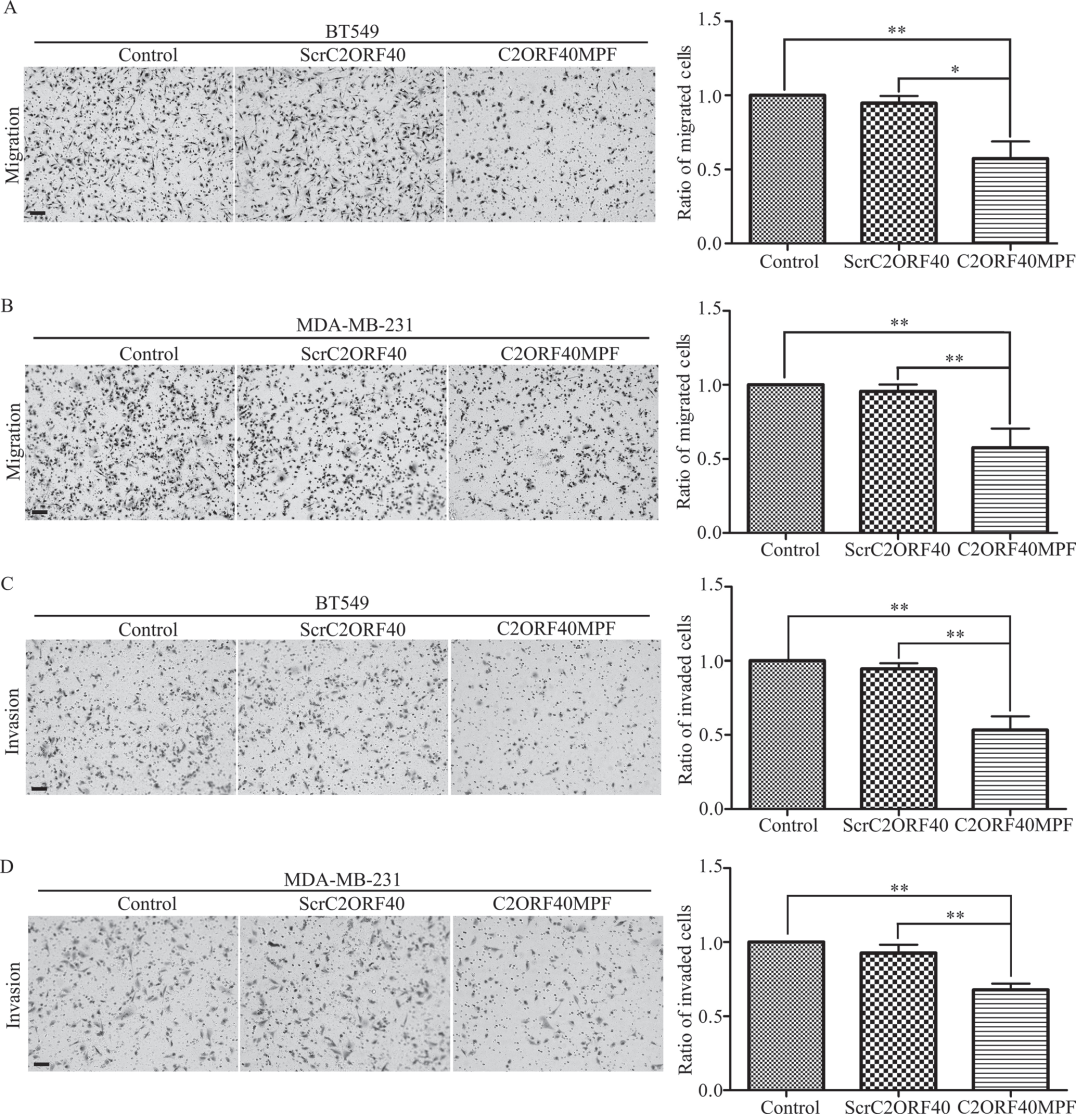
The function of C2ORF40MPF on the motilities of breast cancer cells (**A** and **B**) Transwell migration analysis showed that BT549 and MDA-MB-231 cells treating with C2ORF40MPF possessed less migrating abilities. (**C** and **D**) Matrigel invasion assays showed that BT549 and MDA-MB-231 cells treating with C2ORF40 mimic peptide possessed less invading abilities. Scale bar, 200 μm. All results are from at least three independent experiments. *P < 0.05 and **P < 0.01 based on the Student *t* test. Data are represented as mean ± SD.

In our previous report, we have shown that stable restoration of C2ORF40 expression could suppress the migration and invasion of human breast cancer cells [5]. In the present study, we then evaluated the effect of C2ORF40MPF on the migration and invasion of breast cancer cells. The results indicated that C2ORF40MPF inhibits the mobility of BT549 cells compared with non-treatment control or ScrC2ORF40 (Figure 3A) by transwell assay. The similar results were found in MDA-MB-231 cells (Figure 3B). Moreover, matrigel chamber assay was used to evaluate the effect of C2ORF40MPF on cell invasion. As shown in Figure 3C and 3D, C2ORF40MPF significantly inhibited the invasion of BT549 and MDA-MB-231 cells. Collectively, these results indicated that C2ORF40MPF inhibited migration and invasion of human breast cancer cells.

### C2ORF40 and C2ORF40MPF induce mitotic phase arrest in the breast cancer cells

**Figure 4:**
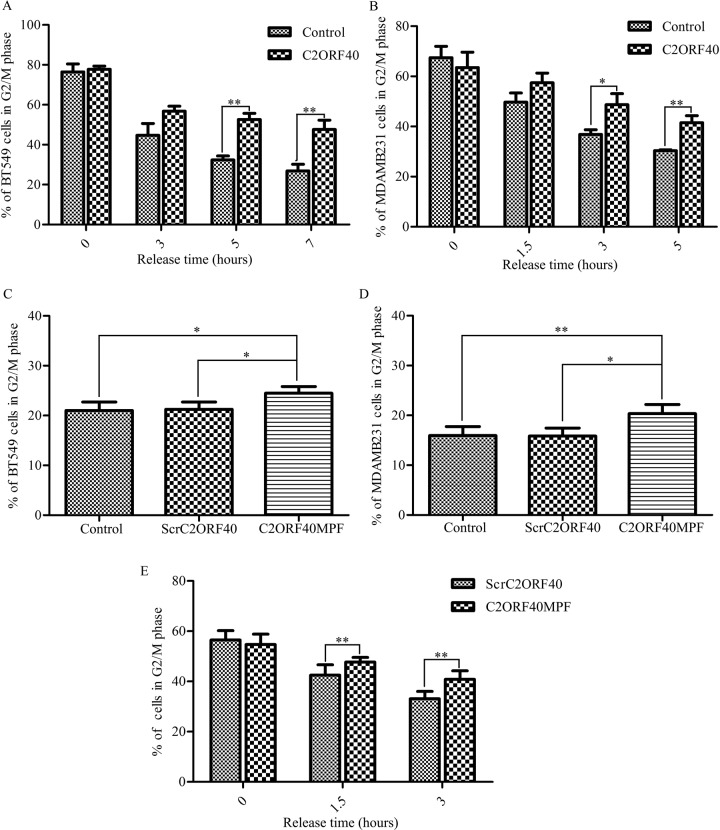
C2ORF40 and C2ORF40MPF induce mitotic phase arrest in the breast cancer cells (**A** and **B**) BT549 and MDA-MB-231 cells with stable restoration of C2ORF40 expression were harvested at the indicated time points after release from synchronization with thymidine-nocodazole block (TNB) and the cell cycle distribution was analyzed by flow cytometry. (**C** and **D**) Breast cancer cells were treated with control or 100 μM ScrC2ORF40 or 100 μM C2ORF40MPF for 48 hours and cell cycle was analyzed by propidium iodide staining and flow cytometry analysis. Next, we combined synchronization with flow cytometric analysis. The cell cycle of MDA-MB-231 cells, which were released after TNB for the indicated time, were detected by flow cytometric analysis (**E**). *P < 0.05 and **P < 0.01 based on the Student *t* test. All results are from three independent experiments. Data are represented as mean ± SD.

To confirm whether C2ORF40 block mitotic progression in cancer cells [5], we combined cell cycle synchronization techniques with flow cytometry assay to analyze the cell cycle progression. Stable restoration of C2ORF40 expression in BT549 and MDA-MB-231 cells were confirmed by RT-PCR and Western blotting (Supplementary Figure 1B and 1C). As shown in Figure 4A and 4B, the fraction of control cells in the G_2_/M phase of cell cycle declined rapidly, whereas the fraction in the C2ORF40 overexpressed cells proceeded significantly slower. Next, we investigated whether C2ORF40MPF has an equivalent effect as C2ORF40 on the cell cycle regulation. Flow cytometry assay results showed that, compared with control or ScrC2ORF40, C2ORF40MPF could increase the G_2_/M phase proportion in BT549 (Figure 4C) and MDA-MB-231(Figure 4D). To further confirm this observation, we combined cell synchronization with flow cytometric analysis. As shown in the Figure 4E, C2ORF40MPF could make the fraction of control cells in the G_2_/M phase of cell cycle declining slower. As cell synchronization with thymidine-nocodazole block could arrest cells in early mitosis [26, 27]. These results indicated that the cell cycle progression was arrested at mitotic phase. In addition, immunofluorescence microscopy analysis showed that C2ORF40 could induce an increase in the percentage of prometaphase (Supplementary Figure 3A and 3B) and generate more multinucleated cells (Supplementary Figure 3C and 3D).

### C2ORF40MPF inhibits tumor growth *in vivo*

**Figure 5:**
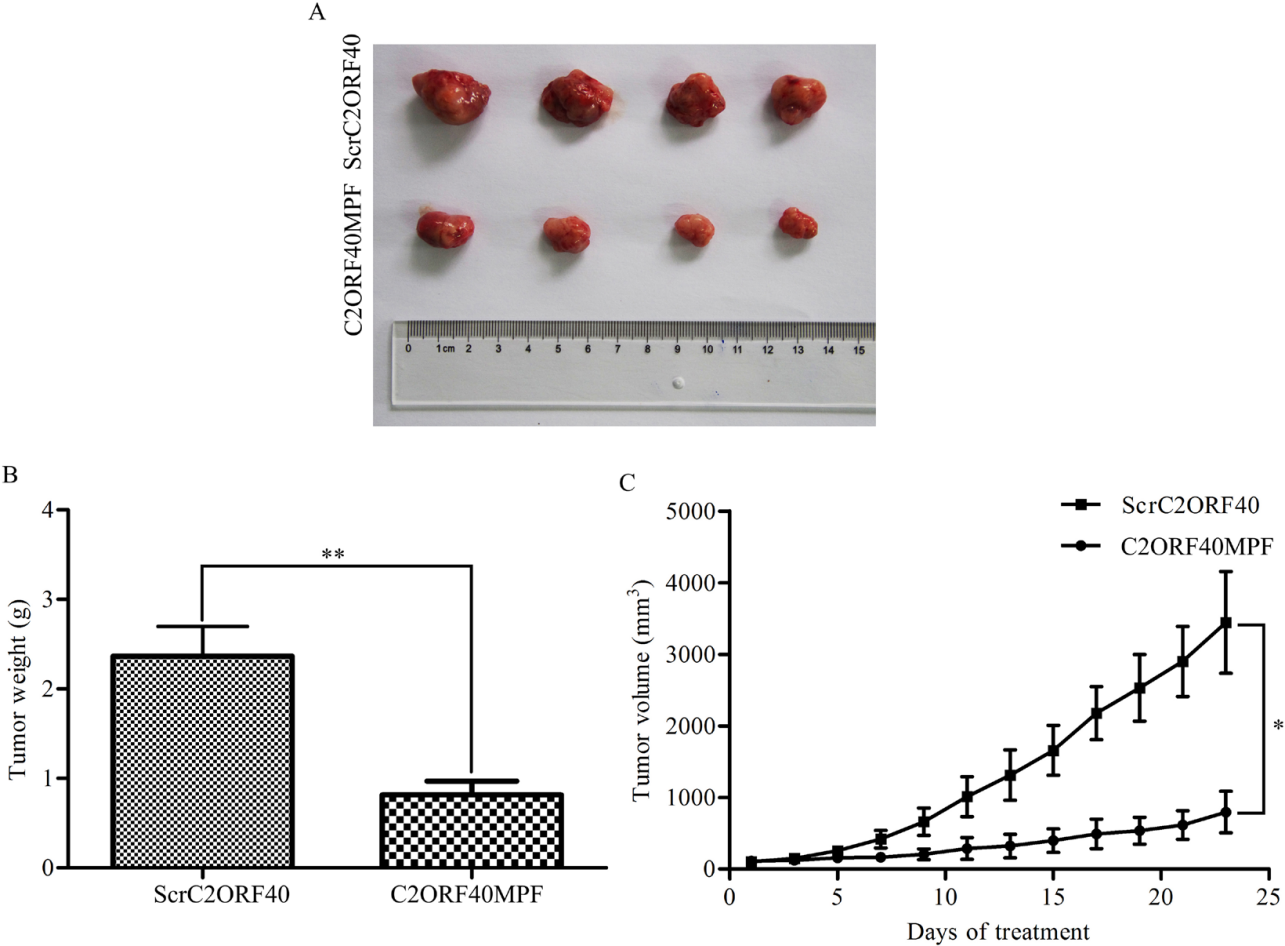
C2ORF40MPF inhibits tumor growth in nude mice MDA-MB-231 cells were subcutaneously injected into the left oxter of nude mice. When the tumor volume reached 75 to 100 mm^3^, mice were treated with 30 mg/Kg ScrC2ORF40 or C2ORF40MPF per day for 23 days. At the end of the study, the tumors (**A**) were taken for pictures. (**B**) The measurement of the tumor weight. (**C**) The tumor volume of nude mice were calculated and recorded every two days. *n* = 4 for each group.*P < 0.05 and **P < 0.01 based on the Student *t* test. Data are represented as mean ± SD.

To evaluate the effects of C2ORF40MPF *in vivo*, a xenograft model was established in nude mice. For the xenograft model, 3 × 10^6^ MDA-MB-231 cells were subcutaneously injected into the left oxter of nude mice. When the tumor volume reached 75 to 100 mm^3^, mice were treated with C2ORF40MPF once per day for 23 days. The final results indicated that C2ORF40MPF could significantly inhibit the tumor growth (Figure 5A) and induce a decrease in tumor weight (Figure 5B) and volume (Figure 5C). These results suggested that C2ORF40MPF could serve as a potential therapeutic treatment for breast cancer growth.

## DISCUSSION

In recent years, investigators have found that C2ORF40 encodes a secreted protein which would be cleaved to generate soluble peptides by proteolytic processing [17, 18] and this proteolytic processing is required to exert its cell-type specific tumor suppressing function [19, 28]. However, whether the C2ORF40-derived peptide exerts a potential therapeutic role in breast cancer has not been reported. In the current study, we synthesized a short 16 amino acid peptide (C2ORF40MPF) as the sequence of thrombin processed human C2ORF40 and explored the biological function of this small peptide in breast and lung cancer cells. For the first time, we demonstrated that this mimic peptide (C2ORF40MPF) could suppress cell proliferation and invasion of breast and lung cancer cells *in vitro* and inhibit the tumor growth in the xenograft mice model, indicating that this mimic peptide has therapeutic potentials.

C2ORF40 has been identified as a tumor suppressor gene in various human cancer types including esophageal [10, 11], colorectal [2], breast [29] and glioma cancers [4]. Downregulation of C2ORF40 in cancer cell lines and clinical tissues was found mainly due to its promoter hypermethylation and correlates with TNM stage, metastasis and patient survival [2, 7, 12, 30–33]. A large body of evidence has shown that restoration of C2ORF40 expression could inhibit cancer cell proliferation, migration and invasion *in vitro* and *in vivo* [5, 34–37]. These data collectively indicate that restoration of C2ORF40 expression may be of clinical therapy values for some malignant carcinomas.

Reactivation of gene expression *in vivo* is still a challenge. In addition, demethylating treatment was reported not to recoup C2ORF40 expression in some prostate cell lines [9]. Exogenous administration of active C2ORF40 protein or peptide would be an alternative choice. Unlike traditional tumor suppressor genes, the C2ORF40 gene product is a secreted protein with furin-like and thrombin cleavage sites [19, 28, 38]. Thus, this protein could be cleaved into multiple peptides by the post-translational proteolytic processing [17, 19]. Indeed, C2ORF40-derived peptides were detected in the culture medium of cells overexpressing C2ORF40 [2, 21, 38] and this processing is proved to be required for C2ORF40 to exert its tumor suppressing function [22]. However, whether the C2ORF40 derived peptides have potential therapeutic efficacy in human breast cancer has rarely been investigated.

Researchers have confirmed a peptide itself derived from thrombin-processed C2ORF40 could induce the myeloid cell accumulation and activate macrophages to exert the proinflammatory function in glioma [38], [39]. In the present study, we synthesized a short peptide to mimic the thrombin-processed C2ORF40 and analyzed if this short synthetic peptide could have tumor suppressing function in breast cancer. Our results showed that administration of this short synthetic peptide (C2ORF40MPF), like overexpression of C2ORF40, could inhibit breast cancer cell proliferation, migration and invasion *in vitro* and suppress tumor growth in breast cancer xenograft models. Cell cycle analysis further confirmed that this C2ORF40MPF might suppress cancer cell growth through inducing G_2_/M phase arrest, which was consistent with the function of C2ORF40 overexpression as we reported here and previously [5]. Our data demonstrated that this short synthetic peptide could mimic the function of C2ORF40 to inhibit breast cancer by inducing cell cycle arrest at G_2_/M phase. Taking into consideration that this peptide would be more stable by its small molecular weight with only sixteen amino acids, this synthetic peptide provides advantages over ectopic C2ORF40 expression in therapeutic means.

In summary, we delineated for the first time that a C2ORF40 mimic peptide fragment (C2ORF40MPF) could attenuate breast cancer tumorigenesis *in vitro* and *in vivo*. Taking the advantage of the size and the nature of its natural existence *in vivo*, our results suggest that this short synthetic peptide fragment (C2ORF40MPF) of human C2ORF40 may be a potential therapeutic agent of human breast cancer and other cancers.

## MATERIALS AND METHODS

### Western blotting analysis

For Western blotting, 30 μg of protein extracts per lane were electrophoresed with denaturing SDS-polyacrylamide gels and transferred to PVDF membranes (Millipore). The membranes were blocked 5% BSA (Bovin Serum Albumin) for 1 hour at room temperature, incubated with primary antibodies (C2ORF40, Santa Cruz Biotechnology and GAPDH, Abcam) overnight and then washed three times with TBST followed by incubation with their respective secondary antibodies. We visualized the signal using ECL detection reagent (Millipore). Quantification of the western was based on the fold changes of band densities calculated by Image j software and normalization to GAPDH expression.

### RT-PCR and quantitative real-time RT-PCR

RT-PCR and quantitative real-time RT-PCR were performed as described previously [5, 40]. TRIzol Reagent was purchased from Invitrogen Life Technologies. One microgram of total RNA was reverse transcribed by a First Strand Synthesis kit (Fermentas). RT-PCR and quantitative real-time RT-PCR (qRT-PCR) were performed to measure the expression of *C2ORF40* and *GAPDH*. *GAPDH* was used as an internal control gene. Quantification of qPCR was based on the CT method and normalization to *GAPDH* expression. The primers of *C2ORF40*: forward primer 5′-GGTACCAGCAGTTTCTCTACATG-3′ and reverse primer 5′-CAGCGTGTGGCAAGTCATGGTTAGT-3′; the primers of *GAPDH*: forward primer 5′-GCCGCATCTT CTTTTGCGTCGC-3′ and reverse primer 5′-TCCCGTT CTCAGCCTTGACGGT-3′.

### Clinical specimen collection

Seventy breast cancer patients undergoing surgical resection at Qilu Hospital of Shandong University (Jinan, China) between February 2014 and January 2015 were included in the present study. We obtained seventy paired fresh tumor tissues and tumor adjacent tissues and the tissue samples were kept at −180°C liquid nitrogen freezers before use. Final pathologic diagnosis of all the specimens was confirmed by pathologists in Qilu Hospital of Shandong University. The experimental protocols were approved by patients’ signed consent and the institutional review committee. In addition, we purchased a breast tissue array, with 89 tumor tissues and 10 normal breast tissues, from Alenabio, China BC081116C. Pathologic tumor-node-metastasis (pTNM) staging is based on the 7th staging classification of AJCC/UICC(2010).

### Immunohistochemical staining and scoring

Immunohistochemical (IHC) staining was carried out as previously described [41]. The IHC analysis of C2ORF40 was performed on the breast array from Alenabio, China BC081116C. We blocked the endogenous peroxidase activity with 3% hydrogenperoxide. Antigen retrieval was carried out in Tris-EDTA buffer (pH-9.0) for 20 minutes at more than 92°C. Tissue sections were incubated with the primary antibody for 16 hours at 4°C, then incubated for 30 minutes at 37°C and subsequently with a secondary biotinylated antibody (SP-9000, China) for 30 minutes at 37°C. After that, the tissue sections were incubated with the streptavidin–peroxidase complex for 5 minutes. The immunohistochemical scoring system was evaluated by a combination of the extent and intensity of staining as previously described [42].

### Cell culture and peptide synthesis

BT549, MDA-MB-231, A549 and H1299 cells were purchased from American Type Culture Collection and cell culture was according to the manufacturer's protocol. All the cell lines were maintained at 37°C in a 5% CO_2_ atmosphere. We revived the cell lines every 3 to 4 months. The following peptides were synthesized by Synpeptide (Shanghai, China): C2ORF40 mimic peptide fragment (C2ORFMPF), SPYGFRHGASVNYDDY; Scrambled C2ORF40 mimic peptide (ScrC2ORFMPF), DAFYYRNGDHYPVSGS.

### MTT assay

Cell proliferation was analyzed by MTT (3-[4, 5-dimethylthiazol-2-yl]-2, 5-diphenyltetrazolium bromide, Solarbio, M8180) assay. BT549, MDA-MB-231, A549 and H1299 cells were seeded in 96-well plates at the density of 3 × 10^3^ cells and treated with scrambled C2ORF40 mimic peptide or C2ORF40 mimic peptide. At each time point (24, 48, 72 hours), 10 μl of 5 mg/ml MTT (Thiazolyl Blue) solution was added to each well and incubated for 4 hours at 37°C. After removing cell medium carefully, 150 μl DMSO were dropped and OD values were measured by the machine (Multiskan 3).

### Colony formation assay

For the colony formation assay, 600 cells were seeded into the 60 mm dishes and treated with 100 μM scrambled C2ORF40 mimic peptide or 100 μM C2ORF40 mimic peptide. Cells were incubated in a homogeneous atmosphere with 5% CO_2_ at 37°C for two weeks. Then methal alcohol was used to fix the clones for 20 minutes and Giemsa (Sigma, GS-500) was used to stain the clones for 30 minutes. Lastly we counted the clones under a microscope.

### Migration assay

For the migration assay, BT549 and MDA-MB-231 cells were seeded into 60 mm dishes and incubated with control or 100 μM scrambled C2ORF40 mimic peptide or 100 μM C2ORF40 mimic peptide. After 24 hours, 3 × 10^4^ cells were seeded onto the upper chamber (BD, 3097) with control or 100 μM scrambled C2ORF40 mimic peptide or 100 μM C2ORF40 mimic peptide and 10% fetal bovine serum were added to the bottom chamber well. The cells were incubated for 24 hours and fixed with methanol for 30 minutes. Then the cells were stained with Giemsa for 1 hour and the cells on the top surface of membrane were removed by cotton swab. Five random field images were obtained by Olympus IX70 inverted microscope and the number of migrated cells were counted.

### Invasion assay

For the invasion assay, BT549 and MDA-MB-231 cells were cultured as the migration does. After 24 hours, to perform invasion assay, we coated the chambers (BD, 3097) with matrigel (BD, 354605) and incubated them at 37°C for 30 minutes. Then, 3 × 10^4^ cells were seeded into the chamber with control or 100 μM scrambled C2ORF40 mimic peptide or 100 μM C2ORF40 mimic peptide and 10% fetal bovine serum were added to the bottom chamber well. The cells were incubated for 48 hours and fixed with methanol for 30 minutes. Then the cells were stained with Giemsa for 1 hour and the non-invaded cells on the top surface of membrane were removed by cotton swab. The images were obtained by Olympus IX70 inverted microscope and the number of invaded cells were counted.

### Cell cycle analysis

BT549 and MDA-MB-231 cells were plated into 60 mm dishes and incubated with control or 100 μM scrambled C2ORF40 mimic peptide or 100 μM C2ORF40 mimic peptide. After 48 hours, cells were harvested by trypsinization and then fixed by 75% ice-cold ethanol. The fixed cells were washed twice with ice-cold PBS and incubated at 37°C for 30 minutes in 0.5 ml PBS solution containing 20 μg/ml RNase A (Fermentas), then stained with 20 μg/ml of propidium iodide (Sigma-Aldrich) at room temperature for 10 minutes. DNA content was analyzed by flow cytometry assay. The percentages of G_1_ cells, S cells and G_2_/M cells were determined by BD FACS Calibur (Becton Dickinson) and data were analyzed with ModFit software (Verity Software House).

### Cell synchronization

For cell synchronization with thymidine-nocodazole block, cells were grown with 2 mM thymidine (Sigma) for 24 hours, released into fresh medium for 3 hours, and then blocked with the 100 ng/ml nocodazole (Sigma) for 12 hours to arrest cells in early mitosis [26, 27]. Cells were washed with PBS twice, either harvested immediately or transferred into fresh medium for the indicated time and then harvested.

### Tumor growth analysis *in vivo*

Female nude mice (6–7 weeks old) were purchased from Beijing Vitalriver and maintained in microisolator cages. All animals were used in accordance with institutional guidelines and the current experiments were approved by the Use Committee for Animal Care. Xenograft tumor models were established by subcutaneous injection of 3 × 10^6^ MDA-MB-231 cells into the left oxter of nude mice. When the tumor volume reached 75 to 100 mm^3^, mice were divided into two groups with 4 animals in each group. The mice were treated with 30 mg/kg Scrambled C2ORF40 mimic peptide or C2ORF40 mimic peptide by intratumoral injection once per day. The tumors and the weight of mice were measured every 2 days for 23 days. The tumor volumes (V) of nude mice were calculated by equation: V = (length × width^2^)/2.

### Statistical analysis

Data were described as mean ± standard deviation (SD). Comparisons between different groups were performed where indicated with the Student two-tailed *t* test. *P* < 0.05 was considered to indicate the criterion of statistical significance. Statistical analysis was carried out with SPSS/Win11.0 software (SPSS Inc).

## SUPPLEMENTARY FIGURES


