# Supplementary material for: A short synthetic peptide fragment of human C2ORF40 has therapeutic potential in breast cancer
Source: Oncotarget. 2017 Mar 30;8(26):41963–74. doi: 10.18632/oncotarget.16713 (PMC5522041; doi:10.18632/oncotarget.16713)
Supplement: Supplementary file 1 [file oncotarget-08-41963-s001.pdf]

A short synthetic peptide fragment of human C2ORF40 has therapeutic potential in breast cancer

Supplementary Materials

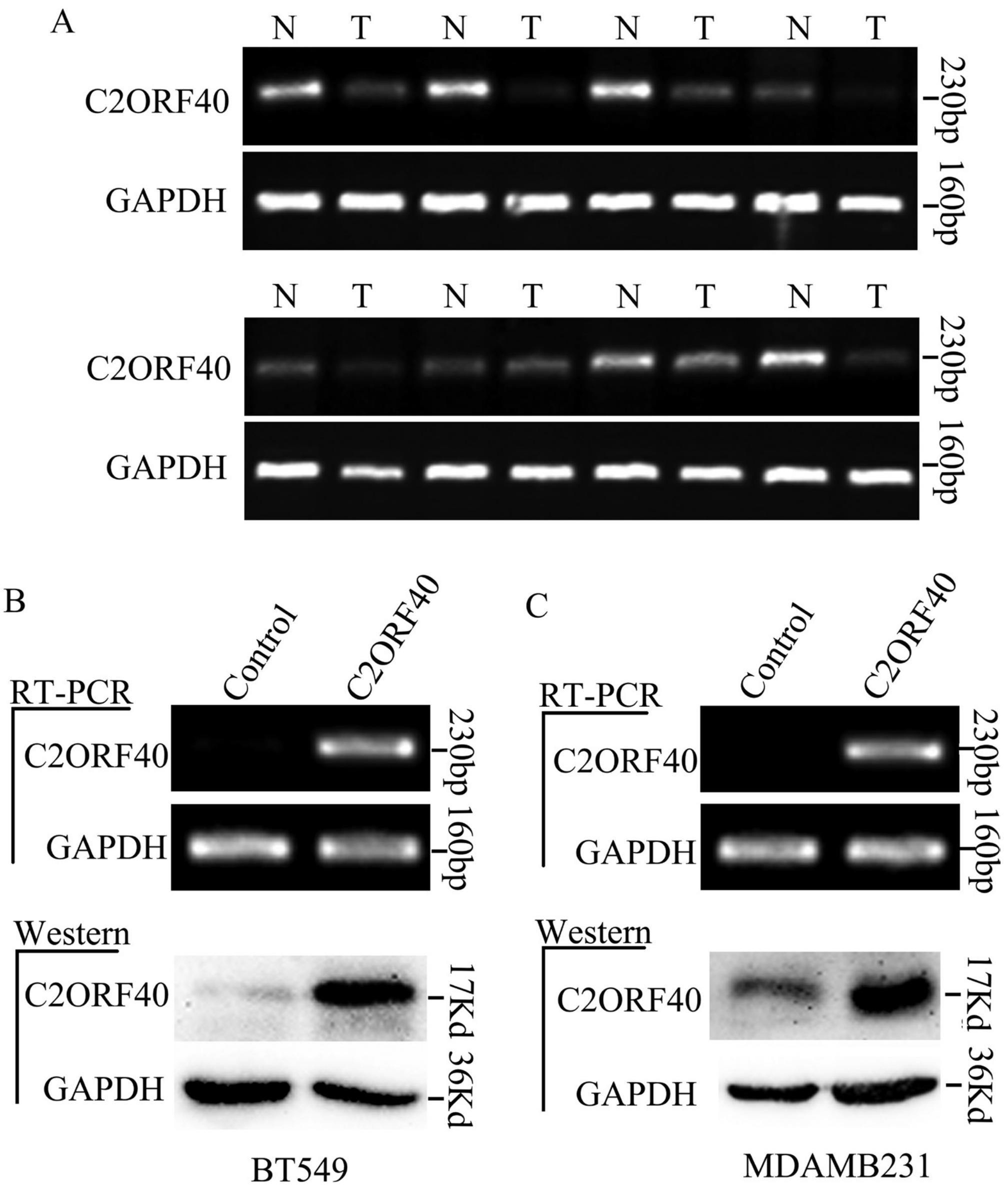

Supplementary Figure 1: (**A**) RT-PCR analysis of C2ORF40 mRNA level in human breast cancer tissues and adjacent non-cancerous tissues. Representative results were shown. GAPDH protein was used as the control. N, adjacent non-cancerous tissues; T, human breast cancer tissues. C2ORF40 expression in retroviral transduced (**B**) BT549 and (**C**) MDA-MB-231 cells was determined by RT-PCR and Western blotting. The results are from three independent experiments. GAPDH mRNA and protein were served as loading controls.

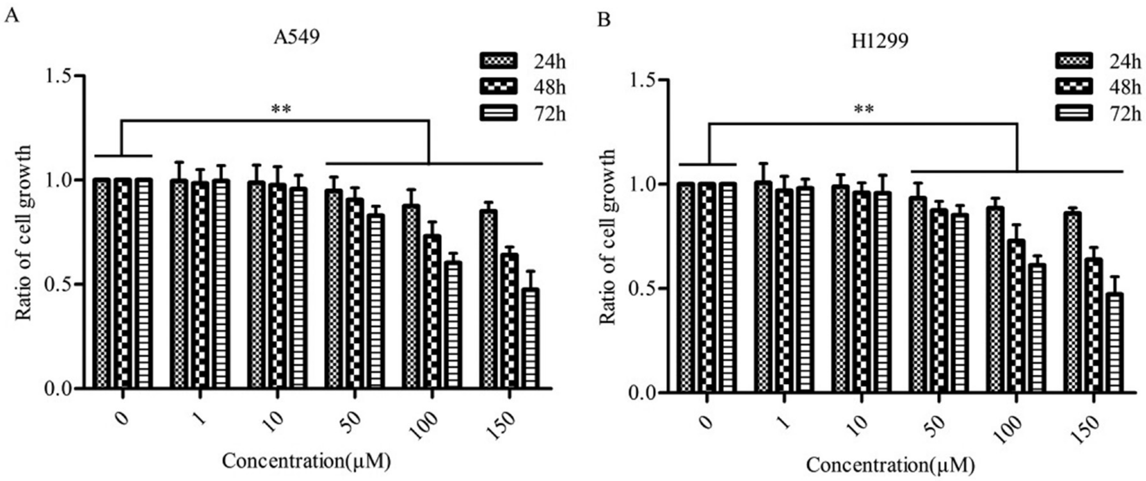

Supplementary Figure 2: The effect of C2ORF40MPF on the proliferation of human lung cancer cells. (**A**) A549 and (**B**) H1299 cells were treated with the indicated concentrations of C2ORF40MPF for 24, 48, and 72 hours. Then, cell proliferation were examined by MTT assay. The results are from three independent experiments. $^{**}P < 0.01$ based on the Student *t* test. Data are represented as mean ± SD.

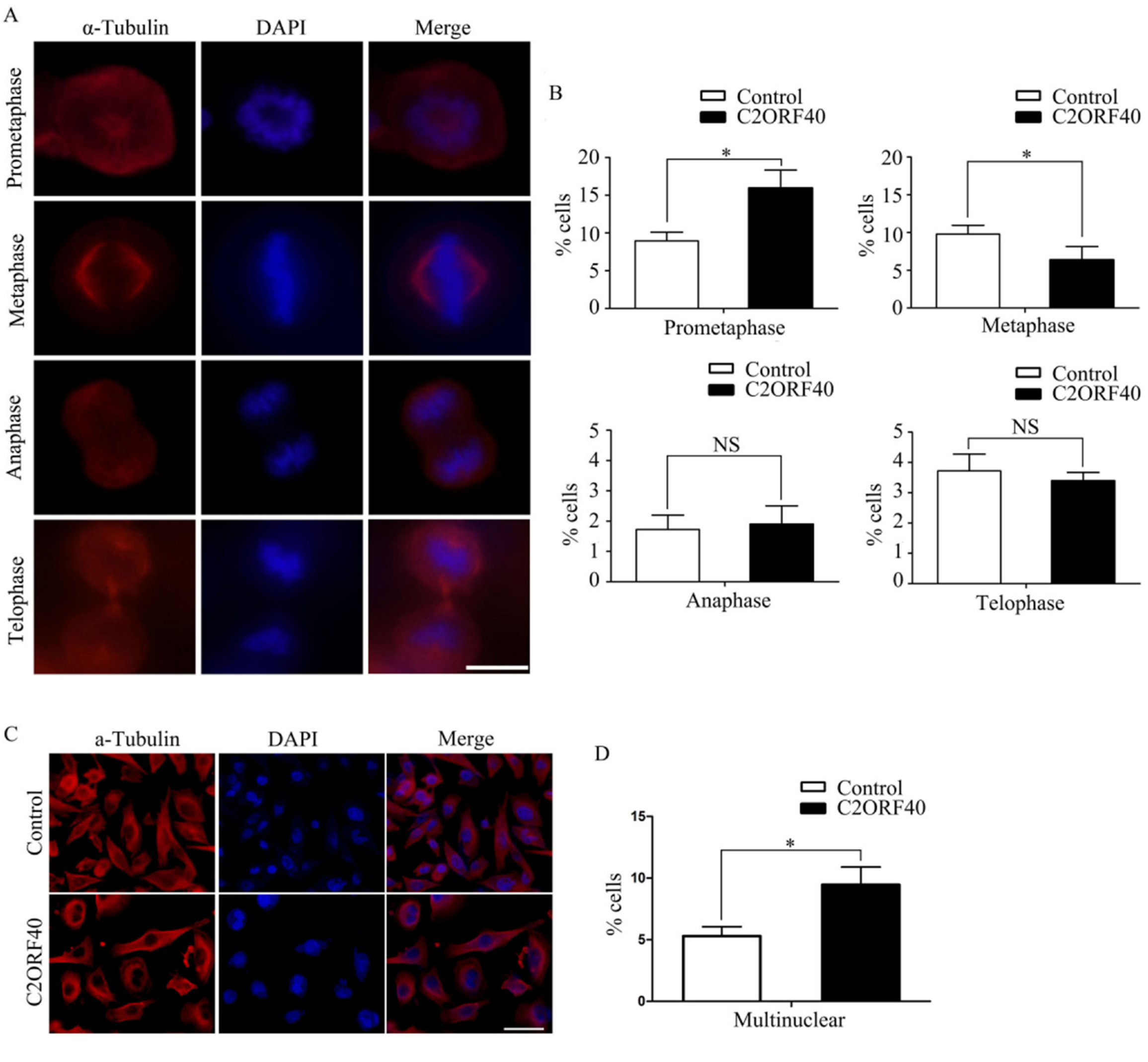

Supplementary Figure 3: C2ORF40 delays cell cycle progression of breast cancer cells at prometaphase of mitotic phase. (**A**) Asynchronously growing BT549 cells are stained for DAPI (blue) and α-tubulin (red) by immunofluorescence assay. Representative cells from different stages of mitotic phase are shown. (**B**) Cells which were released after thymidine-nocodazole synchronization for five hours were scored for prometaphase, metaphase, anaphase and telophase of mitosis ($n \geqq 100$). (**C**) Asynchronously growing BT549 cells were immunostained with anti-α-tubulin antibodies. (**D**) More than 100 cells per sample were scored for the presence of multinucleation (≧ 2 nuclei/cell). Scale bars, 10 µm (A) and 50 µm (D). $*P < 0.05$; $**P < 0.01$; NS, not significant based on the Student t test. All results are from three independent experiments. Data, mean ± SD.

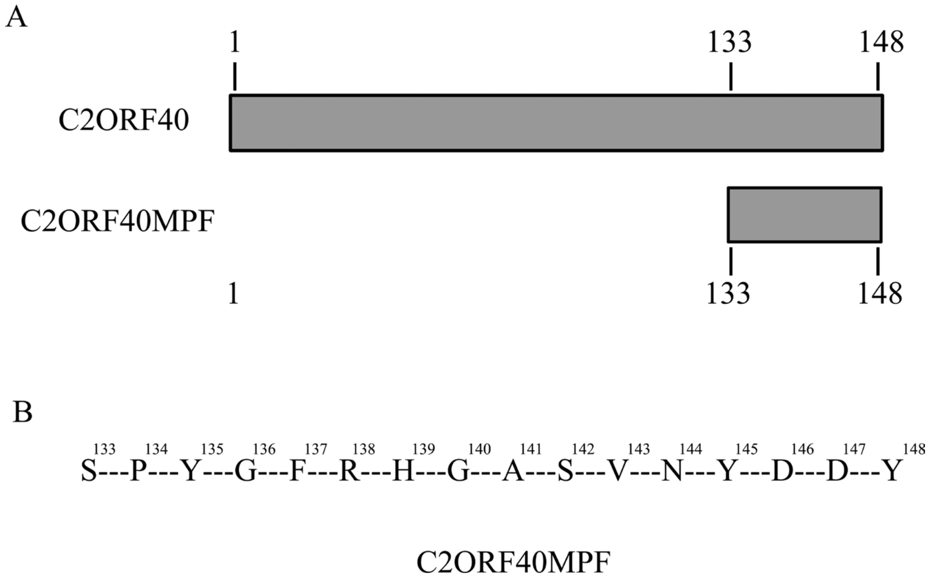

Supplementary Figure 4: Localization of C2ORF40MPF in C2ORF40 protein and the peptide sequence. (**A**) Overview of the localization of C2ORF40MPF in C2ORF40 protein. (**B**) The peptide sequence of C2ORF40MPF.
